# P-1422. Low Use of Oral Antibiotics for People Hospitalized with Serious Injection-Related Infections: the CHOICE+ Cohort

**DOI:** 10.1093/ofid/ofae631.1597

**Published:** 2025-01-29

**Authors:** Hannah E Flores, Habib Omari, Meghan Derenoncourt, Jasmine Stevens, Ishan Kumar Vaish, Sumitha Raman, Ayako Wendy Fujita, Joseph E Carpenter, Jillian S Catalanotti, Sarah Kattakuzhy, Alaina Steck, Irene Kuo, Becky Reece, Elana S Rosenthal, Edward C Traver

**Affiliations:** University of Maryland, Baltimore, Maryland; University of Maryland Baltimore, Baltimore, Maryland; University of Maryland, Baltimore, Baltimore, Maryland; University of Maryland School of Medicine, Baltimore, Maryland; University of Maryland Medical School, Baltimore, Maryland; George Washington University, Washington, District of Columbia; Emory University School of Medicine, Atlanta, Georgia; Emory University School of Medicine, Atlanta, Georgia; The George Washington University of Medicine and Health Sciences, Washington, District of Columbia; Institute for Human Virology (IHV), University of Maryland School of Medicine, Baltimore, Maryland; Emory University, Atlanta, Georgia; George Washington University Milken Institute School of Public Health, Washington, District of Columbia; West Virginia University, Morgantown, WV; Institute for Human Virology (IHV), University of Maryland School of Medicine, Baltimore, Maryland; University of Maryland School of Medicine, Baltimore, Maryland

## Abstract

**Background:**

Oral (PO) antibiotics are a convenient alternative to parenteral antibiotics and decrease the need for inpatient care. Increasing evidence shows PO antibiotics are effective even in severe infections. We aim to assess PO antibiotic use among hospitalized people who inject drugs (PWID).

**Figure:**
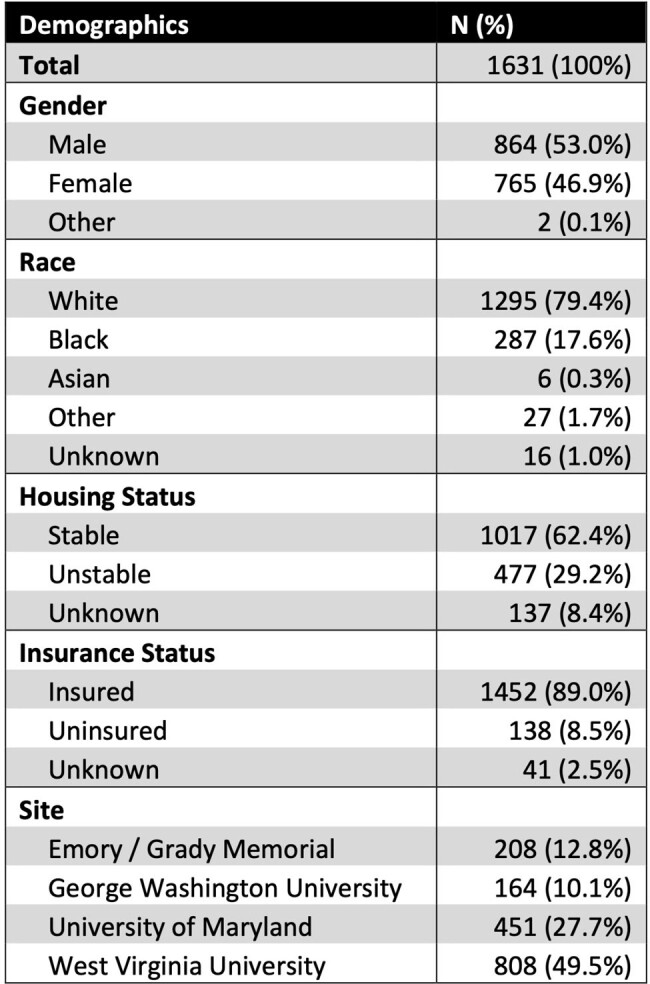

**Methods:**

CHOICE+ is a multisite retrospective cohort study of adults hospitalized at four healthcare systems with infections resulting from injection opioid use between 1/1/2018 and 3/31/2022. Data were collected by abstraction of the electronic medical record. Patients were categorized by intended route of the antibiotic course. Data were analyzed by the chi-square test, Fisher’s exact test, and multivariate logistic regression.

**Figure:**
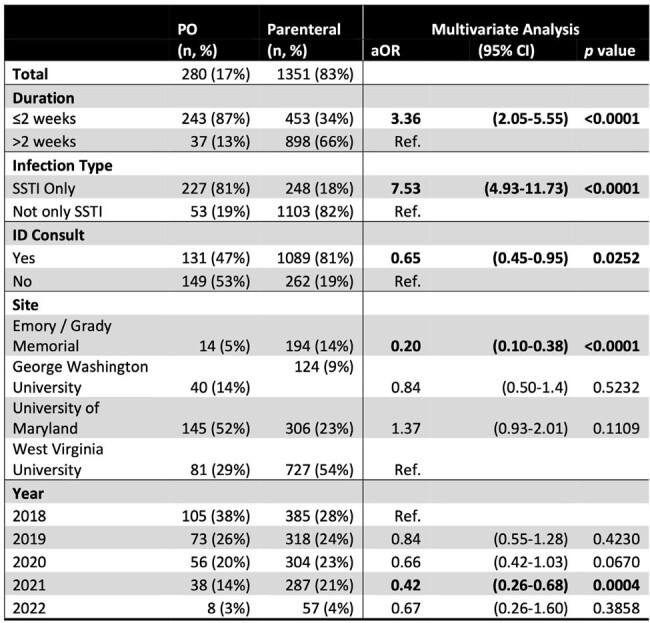

PO, oral; SSTI, skin and soft tissue infection.

**Results:**

1631 patients had an intended treatment plan with either PO (17%) or parenteral (83%) (intravenous, long-acting infusion, or other) antibiotics (Table 1). There was no difference in PO antibiotic use based on gender or unstable housing. PO antibiotics were more likely to be used when planned antibiotic duration was ≤2 weeks (p< 0.0001) and when only skin and soft tissue infection (SSTI) was present (p< 0.0001, Table 2). Alternatively, infectious disease consultation was associated with lower use of PO antibiotics (p< 0.0001). From 2018 to 2022, PO antibiotic use decreased (p< 0.0001, chi-square for trend, Figure 1). Those treated with PO antibiotics were less likely to have ID follow-up scheduled (p=0.0002) and less likely to attend ID follow up (p=0.0024, Figure 2).

**Figure:**
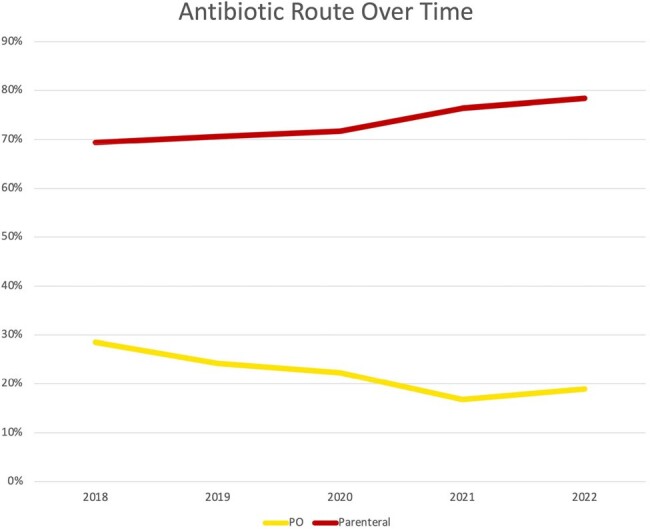

PO, oral.

**Conclusion:**

PO antibiotics were rarely used in this large multisite cohort of PWID, especially for infections other than SSTI. Surprisingly, use decreased over the study period, despite emerging evidence for PO antibiotics in complex infections for people who do not inject drugs. PWID with complex infections require long courses of antibiotics, but face barriers to prolonged hospitalization and outpatient infusions due to underinsurance, unstable housing, and untreated addiction. PO antibiotics may facilitate antibiotic completion while reducing patient-directed discharge, and therefore are likely underutilized for PWID.

**Figure:**
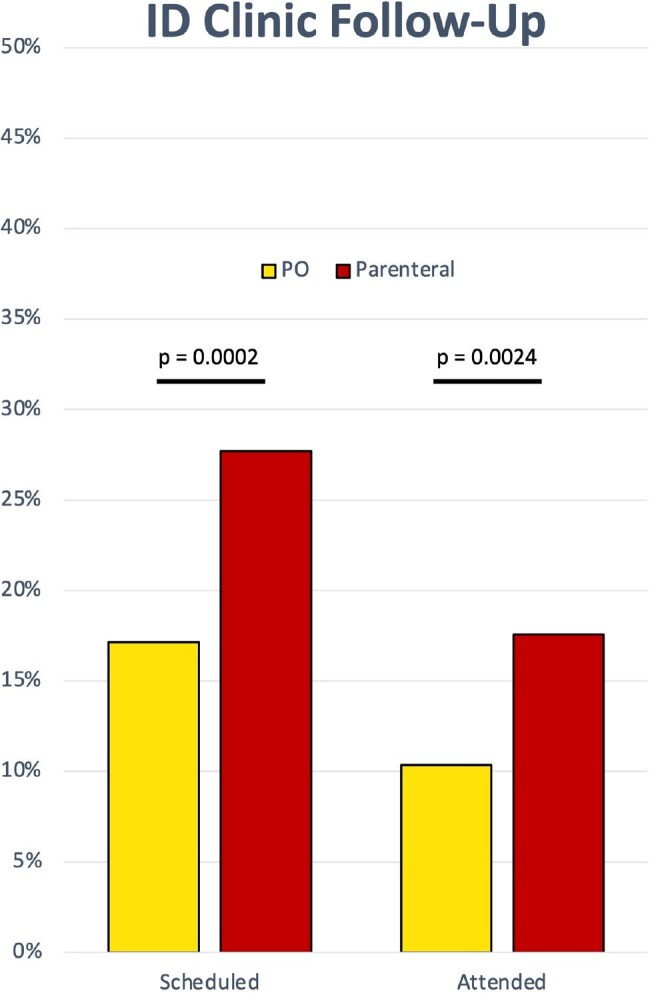

PO, oral.

**Disclosures:**

**Elana S. Rosenthal, MD**, Gilead Sciences: Grant/Research Support|Merck: Grant/Research Support

